# Bovine serum albumin nanoparticles encapsulating Dasatinib and Celecoxib for oral cancer: Preparation, characterization, and *in-vitro* evaluation

**DOI:** 10.1007/s00210-025-03829-1

**Published:** 2025-02-12

**Authors:** Ghadeer AbouBakr Aly, Sally A. Sabra, Medhat Haroun, Maged W. Helmy, Nermine Moussa

**Affiliations:** 1 Department of Biotechnology, Institute of Graduate Studies and Research, Alexandria University, Alexandria, Egypt; 2 Department of Pharmacology and Toxicology, Faculty of Pharmacy, Damanhur University, Damanhur, Egypt

**Keywords:** Oral squamous cell carcinoma, Dasatinib, Celecoxib, Bovine serum albumin nanoparticles, Src/ Focal Adhesion Kinase** (**FAK) axis, Cyclooxygenase-2** (**COX-2)/ Prostaglandin E2 (PGE2) axis

## Abstract

Oral squamous cell carcinoma is a diverse complex disease. Despite the ever-expanding repertoire of anti-cancer treatments, the outcomes are often inadequate highlighting the urgent need for innovative approaches. In this regard, co-targeting signaling pathways such as Src and COX-2 have attracted growing attention in several cancers, but co-inhibition of these two pathways using dasatinib and celecoxib has not been explored in oral cancer. However, the therapeutic efficacy of these drugs is limited due to their low aqueous solubility. Nanoencapsulation can improve this by utilizing naturally available proteins due to their ease of fabrication and biocompatibility. In this sense, this study aimed at preparing and characterizing dastatinib (DAS)/celecoxib (CXB)-loaded bovine serum albumin (BSA) nanoparticles as well as investigating their potential anticancer effects in vitro on SCC-4 oral cancer cell line. DAS/CXB-loaded BSA nanoparticles (NPs) were fabricated by the desolvation method, then characterized in terms of their hydrodynamic particle size, zeta potential, morphology and in vitro drug release. The IC50 was determined via the MTT assay. Cyclin D1, COX-2, p-Src and FAK protein expression levels were determined using ELISA while active caspase-3 was determined colorimetrically. DAS/CXB-loaded BSA NPs exhibited particle size of 336.6 ± 1.098 nm with low PDI value of 0.211 ± 0.019 and zeta potential of -35.0 ± 4.03 mV. Moreover, the in vitro cytotoxicity study revealed decreased IC50 value in case of the dual drug-loaded NPs compared to all treated groups, with significant decrease in the expression levels of cyclin D1, COX-2, p-Src and FAK proteins, besides, increased caspase-3 level. The findings suggest that DAS/CXB-loaded BSA NPs could serve as a drug delivery platform with increased antitumor effectiveness.

**Supplementary Information:**

The online version contains supplementary material available at 10.1007/s00210-025-03829-1.

## Introduction

Oral squamous cell carcinoma (OSCC) is a significant global health issue, with more than 389,000 new cases reported each year and an estimated sixty-five percent increase by the year 2050, highlighting the escalating burden of this disease. The incidence varies, with rates ranging from 1.1–9.9 per 100,000 across various regions. The burden is linked to the Human Development Index (HDI), with anticipated increases in incidence for all HDI tiers by 2050: around 147.8 percent in low HDI countries, 94.2 percent in medium HDI countries, 67.3 percent in high HDI countries, and 34.3 percent in very high HDI countries. Men are more affected by OSCC, with peak incidence after the age of 50. Though, there is an alarming rise in cases among women as well as younger individuals, many of whom have not been subjected to traditional risk factors as tobacco and alcohol use (Coletta et al. 2024), underscoring the pressing need for in-depth understanding of the molecular mechanisms governing OSCC’s pathogenesis.

At the core of OSCC’s development is an intricate interplay of genetic mutations as well as epigenetic changes, which interrupt the normal cellular processes and enable malignant transformation. As the disease progresses, malignant cells acquire further genetic and epigenetic changes, leading to invasive and metastatic characteristics. The epithelial-mesenchymal transition (EMT) enables cancer cells to acquire motility and invade neighboring tissues (Georgaki et al. 2021). These molecular modifications pinpoint key therapeutic targets for interrupting the invasive capability of OSCC and highlight the demand for therapies that can inhibit these key tumor progression steps.

Despite the ever-expanding arsenal of anti-cancer therapeutics*,* treatment failure is still an inevitable outcome (Kozar et al. 2019), highlighting the urgent need for innovative approaches. One promising strategy is the dual targeting of vital molecular signaling cascades that initiate OSCC’s progression. This study explores the potentiality of co-targeting Src and cyclooxygenase-2 (COX-2) signaling pathways, both of which are implicated in various stages of oral cancer, from proliferation to metastasis. On one hand, Src family kinases (SFKs) promote OSCC proliferation, invasiveness, as well as metastasis via activating a myriad of down-stream molecular targets (e.g. phosphatidylinositol 3-kinase, signal transducers and activators of transcription protein, and growth factor receptor-bound protein 2 (GRB2)/rat sarcoma viral oncogene (RAS)/V-raf murine sarcoma viral oncogene (RAF) signaling cascades). SFKs facilitate the migration and invasion of OSCC cells via disrupting cell adhesion and enhancing matrix degradation, making Src a fascinating target, as impeding its activity could curb the invasive capacity of OSCC cells (Wang et al. 2020). COX-2, on the other hand, is a crucial signaling cascade in the pathophysiology of OSCC, where it contributes substantially to tumor progression. COX-2 catalyzes the conversion of arachidonic acid into prostaglandin E2 (PGE2). Via binding to its receptors, PGE2 activates signaling cascades which encourage cell proliferation, survival, and migration; promote angiogenesis by triggering endothelial cell growth; and support immune evasion via hampering the functionality of T cells as well as natural killer cells. Accordingly, inhibiting COX-2 activity can ultimately suppress the metastatic capacity of OSCC cells, emphasizing the therapeutic potential of targeting COX-2 (Seyedmajidi et al. 2014). Besides, higher Src and COX-2 activities are evident in OSCC and are associated with: (a) aggressive tumor behavior; (b) therapy resistance; (c) worse prognosis; (d) possibility of lymph node metastasis; and € poor overall- and disease-free survival rates (Karunakaran and Muniyan 2020). Intriguingly, growing evidence suggests that these two pathways exhibit significant cross-talk (Moussa et al. 2021).

Two drugs that hold considerable promise in co-targeting the aforementioned pathways are dasatinib (DAS) and celecoxib (CXB). On one hand, DAS has anti-proliferative, anti-angiogenic, and pro-apoptotic effects on oral cancer cells. It inhibits a myriad of signal transduction cascades (e.g. Src, EGFR, STAT-3, and Mcl-1), crucial in cancer cell proliferation and survival. Dasatinib promotes the apoptotic machinery via the intrinsic caspase pathway and reduces angiogenesis via downregulating the expression of hypoxia-inducible factor 1 alpha (Park et al. 2021; Wei et al. 2024). On the other hand, CXB targets COX-2-mediated pathways that govern cancer progression. It promotes the apoptotic cell death machinery via activating the intrinsic pathway, modulating pro- and anti-apoptotic proteins, and disrupting the mitochondrial function. Celecoxib also inhibits angiogenesis via downregulating the vascular endothelial growth factor and matrix metalloproteinases expression, thereby preventing new blood vessel development within tumors and restricting their utilization of nutrients and oxygen. It reduces inflammation by suppressing COX-2-mediated prostaglandin synthesis, decreasing inflammatory cytokines and chemokines, and regulating immune cell function (Wen et al. 2020). These mechanisms collectively support their potential as therapeutic agents capable of combating OSCC’s progression.

Though no studies have been conducted on DAS/CXB combination in OSCC, previous research has demonstrated promising results in other cancer types. The observed synergistic effect provides a strong rationale for investigating this combination in SCC-4 cells and potentially other OSCC models. DAS/CXB combination allows for simultaneous targeting of different oncogenic pathways and can lead to more effective tumor suppression than either agent alone (Moussa et al. 2021; Lamey et al. 2023).

Despite their promising therapeutic effects, their systemic delivery is fraught with challenges, including toxicity, lack of specificity, and development of drug resistance. One potential solution lies in the use of nanocarrier-based drug delivery systems, which enable active or passive targeting, reduce side effects, improve the pharmacokinetic profile, and allow the simultaneous drug delivery. Natural proteins such as bovine serum albumin (BSA), being safe, affordable, biodegradable, biocompatible, could be an ideal platform in this regard (Lamey et al. 2023; Mustafai et al. 2023). Building on this, this study investigates the preparation and physicochemical characterization of DAS/CXB-loaded BSA nanoparticles and evaluates their anti-cancer effects in vitro using the SCC-4 oral cancer cell line.

## Materials and methods

### Materials

DAS was received as a gift from Apotex Pharma Chem Inc. (Ontario, Canada) and Celecoxib from Borg Pharmaceutical Industries, Egypt. Triton X-100, dimethyl sulfoxide (DMSO), MTT, and BSA were purchased from Sigma-Aldrich (St. Louis, USA). Absolute ethanol was obtained from Merck (Dramstadt, Germany. Fetal bovine serum (FBS) was purchased from PAN Biotech (South America) and Dulbecco’s Modified Eagle Medium (DMEM) form MMS (Lonza Verviers SPRL, Belgium). Glutaraldehyde was purchased fom Loba chemie. (PVT. LTD, India). The rest of the reagents used in the current work were of analytical grade and were used exactly as intended.

### Preparation of DAS/CXB-loaded BSA nanoparticles

DAS/CXB-loaded BSA NPs were fabricated by the desolvation method with slight modifications (An and Zhang 2017). Briefly, DAS and CXB (5 mg of each of them) were co-dissolved in 2 mL absolute ethanol and then the drugs mixture was directly added to an equal volume of aqueous solution of BSA (25 mg/mL) in Milli-Q water (pH 6.9) at room temperature. Afterwards, 40 µL of 8% glutaraldehyde was added to the obtained mixture and the pH of the solution was kept at 7.6. Then, the solution was stirred overnight at 500 rpm using a magnetic stirrer at room temperature. Finally, DAS/CXB-loaded BSA NPs were separated by centrifugation (Mini centrifuge, Mini-14 K, Europe) at 12,000 rpm for 20 min. The collected pellets were then lyophilized using a freeze dryer (Ehrisa beta 1, Germany) and stored in a desicator at 25°C for further analyses. Regarding the blank BSA NPs, they were prepared with the same method except that no drugs were added during the preparation steps (Jahanban-Esfahlan et al. 2022).

### Physicochemical characterization of the DAS/CXB-loaded BSA nanoparticles

Fourier transform infrared (FT-IR) spectroscopy (PerkinElmer, USA) was employed to record the spectrum of each of the DAS/CXB-loaded BSA NPs, blank BSA NPs, free DAS, and free CXB. At 25°C, the spectra were recorded in the 500–4000 cm^−1^ region (Berthomieu and Hienerwadel 2009). Furthermore, the thermograms of DAS/CXB-loaded BSA nanoparticles, blank BSA NPs, free DAS, and free CXB were estimated using differential scanning calorimetry (DSC) (PerkinElmer, USA). Each sample’s dry powder (about 3 mg) was heated in a sealed aluminum pan at 10°C per minute under nitrogen. A plain aluminum pan, as a reference, was used in all runs (Solanki et al. 2021).

To determine the encapsulation efficiency percentage (EE%) of DAS and CXB in the dual-loaded BSA NPs, a high-performance liquid chromatography (HPLC) method was developed. Chromatographic separation was performed using a Shimadsu C8 reversed-phase column (particle size: 5 μm, 4.6 × 150 mm) with an isocratic flow rate of 0.5 mL/min. The mobile phase consisted of acetonitrile and ammonium buffer (pH 11, 45:55 v/v). The concentration of both drugs was measured at 254 nm with a Shimadzu LC-20AD L201148 separation module and a Shimadzu SPD-M20A C209349 photodiode array UV/visible detector. The EE% and the drug loading (DL) percentage were calculated using the following equations:

1 $$EE\% = \frac{Weight\;of\;the\;encapsulated\;drugs\;within\;the\;NPs }{Weight\;of\;total\;drug\;added} \times 100$$

2 $$DL\% = \frac{Weight\;of\;the\;encapsulated\;drugs\;within\;the\;NPs}{Weight\;of\;NPs + weight\;of\;the\;encapsulated\;drugs } \times 100$$

To determine the particle size (PS) as well as the polydispersion index (PDI), a zetasizer (NanoZS/ZEN3600) (Malvern Instruments Ltd., UK) was used. Deionized extra pure water was used to dilute the sample, and backscattering technology was used to measure the PS at a detection angle of roughly 173 °C. At 25 °C, measurements were conducted trice. Before inserting the prepared NPs into the folded capillary cell, they were diluted with 1 mM KCl solution and their zeta potential was determined. The net charge on the NPs surface was determined by utilizing laser Doppler anemometry (Malvern Instruments Ltd., UK) to measure the mean electrophoretic mobility (Chen et al. 2019).

DAS/CXB-loaded BSA NPs were visualized by staining a drop of the diluted sample with uranyl acetate solution, followed by air drying for 30 s, and using a transmission electron microscope (TEM) (JEOL JEM-100S, Tokyo, Japan) for capturing images.

### *In-vitro* drug release study

The study used the dialysis bag technique to track drug release patterns from DAS/CXB-loaded BSA NPs. The release patterns were examined at a pH of 5.5 to mimic the acidic tumor microenvironment. The dialysis bag (12–14 kDa MWCO, Spectra/Pro^®^4 Dialysis Membrane; Spectrum Laboratories, Inc., Rancho Dominguez, CA) was filled with 1 mg of each drug in its free form or a formulation volume equivalent to 1 mg from each drug. Dialysis bags were then submerged in 200 mL of acetate buffer, pH 5 containing Triton X-100 (1%) and kept in a shaking water bath (AZZOTA corporation, USA) at 37°C. This release medium was the FDA-approved dissolution medium for DAS. HPLC was used to determine the percentage of drug release, and the amount of the released drug was determined as follows (Sabra et al. 2019).

3 $$Drug\;release\;\%=\frac{Amount\;of\;drug\;released}{Total\;amount\;of\;the\;encapsulated\;drug}\times100$$

### *In-vitro* cytotoxicity

#### Cell lines and drugs

SCC-4, purchased from the American Type Culture Collection, is an epithelial-like cell derived from the tongue of a fifty-five-year-old male diagnosed with squamous cell carcinoma. DAS and CXB were dissolved in DMSO at the concentration of 10 mM and thereafter stored at 20°C.

#### Cell thawing, Culturing and Sub-culturing of cells

Cell thawing, cell culturing, and sub-culturing were performed as previously described (El-Kishky et al. 2022; Alian et al. 2024).

#### *In-Vitro* cytotoxicity assay

The Microculture Tetrazolium Test (MTT) was used to assess cytotoxicity (Riss et al. 2013). To plate SCC-4 cells, use 200 µL of DMEM with penicillin (one U/mL), streptomycin (1 μg/mL), and FBS (10% v/v). Then, incubation was done at 37°C with 90% air/10% CO_2_. After that, the microtiter plate was maintained in 5% CO_2_ at 37°C for 24 h to enhance cell adhesion. The concentrations of 40 µM, 20 µM, 10 µM, 5 µM, 2.5 µM, and 1.25 µM were used to test DAS, whereas 80 µM, 40 µM, 20 µM, 10 µM, 5 µM, and 2.5 µM were used to test CXB. The concentrations of 40 µM-80 µM, 20 µM-40 µM, 10 µM-20 µM, 5 µM-10 µM, 2.5 µM-5 µM, and 1.25 µM-2.5 µM were the free DAS/CXB combination dosages that were investigated. The same concentrations were used for the DAS/CXB-loaded BSA NPs. The plate was then set aside for 48 h under exactly the same conditions. On day three, 10 µL of MTT reagent was added and incubated for four hours. The supernatant was then carefully removed, and 100 µL of DMSO was added. The plate was left in the darkness for two hours. Each well's optical density (OD) was determined at 570 nm. A dose–effect curve was created to depict the relationship between different drug concentrations on the X-axis and percentage cell viability on the Y-axis. The median inhibitory concentrations (IC50) of DAS and CXB were subsequently quantified using CompuSyn software (version 3.0.1, CompuSyn, Inc. (Yao et al. 2020).

#### Dose Reduction Index (DRI) and Combination Index (CI) Determination

The Dose Reduction Index (DRI) measures the fold decrease in the dosage of an individual drug when utilized in a synergistic combination to accomplish a desired level of efficacy at a reduced dosage compared with using each drug individually (Chou 2010). The Combination Index (CI) was employed to examine the synergistic or antagonistic interactions between the two drugs. The DRI and CI values for DAS/CXB in SCC-4 cells were computed using CompuSyn software (Abdallah et al. 2018; Kabary et al. 2018; Abdelaziz et al. 2019; El-Hanboshy et al. 2021; Abd-Alhaseeb et al. 2022). The CI value ≤ 1 or > 1 denotes synergistic, additive, or antagonistic effects, respectively (Chou 2010).

#### Treatment of SCC-4 cells

SCC-4 cells were separated into four groups including: (a) Control cells; (b) DAS-treated cells; (c) Celecoxib-treated cells; (d) DAS/CXB-loaded BSA NPs-treated cells.

#### Biochemical analyses using ELISA technique

Cyclin D1, COX-2, p-Src, FAK protein expression levels were determined using the following ELISA kits (Novus Biologicals, Inc., USA) (Cat#: NBP2-75100); (Cayman Chemicals, USA) (Cat#: 760151); (R&D Systems, USA) (Cat#: DYC2685-2); (Elabscience, USA) (Cat#: E-EL-H1771); respectively, as per the manufacturer’s guidelines.

In summary, 100 μL of samples or standards were introduced, followed by a 2-h incubation at room temperature. Subsequently, a working solution of 100 μL was added, and the wells were covered and gently agitated to ensure complete mixing. This was followed by an additional incubation for 1 h at 37°C, after which the contents were aspirated and washed three times with approximately 350 μL of 1X wash buffer. Next, 100 μL of detection reagent B working solution was added, and the mixture was incubated for 30 min at 37°C, followed by aspiration and five washes as previously described. Afterward, 90 μL of TMB substrate solution was added to each well and incubated for 20 min at 37°C. Finally, a stop solution of 50 μL was added to each well. The OD was measured using a microplate reader set to 450 nm. A standard curve was generated by plotting the concentrations of the standards on the X-axis against the absorbance on the Y-axis.

#### Determination of Caspase-3 activity in SCC-4 cell lysates

The Caspase-3 assay kit (Sigma Aldrich, USA) (Product Code CASP-3-C) was employed to evaluate Caspase-3 activity in SCC-4 cell lysates. In principle, Caspase-3 can hydrolyze the peptide substrate acetyl-Asp-Glu-Val-Asp p-nitroanilide, resulting in the release of p-nitroaniline (pNA). To conduct the assay, 10 μL of either cell lysates or positive controls were mixed with 1X assay buffer, as per the manufacturer's instructions. Next, 10 μL of a Caspase-3 inhibitor was added, along with 10 μL of the Caspase-3 substrate to initiate the enzymatic reaction. The mixture was incubated for two hours at 37°C, after which the OD was measured at 405 nm. The resulting Caspase-3 activity was quantified based on the formula:

4 $$Activity\;(\mu mol\;pNA/min/ml)=\frac{OD\times d}{\varepsilon\times t\times v}$$

where: OD: observed optical density; ε: pNA absorptivity in mM (10.5); v: Volume of sample in ml; d: Dilution factor; and t: Reaction time in minutes.

### Statistical analysis

The average distribution of the collected data was evaluated through normality testing. All data were expressed as the mean ± standard deviation (SD). Data were statistically analyzed using GraphPad Prism 8.0 (GraphPad Software, CA, USA). A one-way analysis of variance (ANOVA) was followed by a post-hoc Tukey multiple-comparisons test to determine statistical significance. P-values < 0.05 were considered statistically significant. Each experiment was conducted independently three times, with three replicates.

## Results

### Preparation of DAS/CXB-loaded BSA nanoparticles

Using a desolvation procedure, albumin was first coacervated with an organic solvent before being chemically cross-linked with glutaraldehyde to yield DAS/CXB-loaded BSA nanoparticles. Three steps make up the desolvation process: (1) adding a desolvating solution (usually ethanol or acetone) to the protein aqueous solution to get the coacervate; (2) chemical cross-linking to toughen the coacervate; and finally (3) centrifuging and purifying the resultant NPs (Jahanban-Esfahlan et al. 2016).

### Physicochemical characterization of the produced BSA nanoparticles

**Fig. 1:**
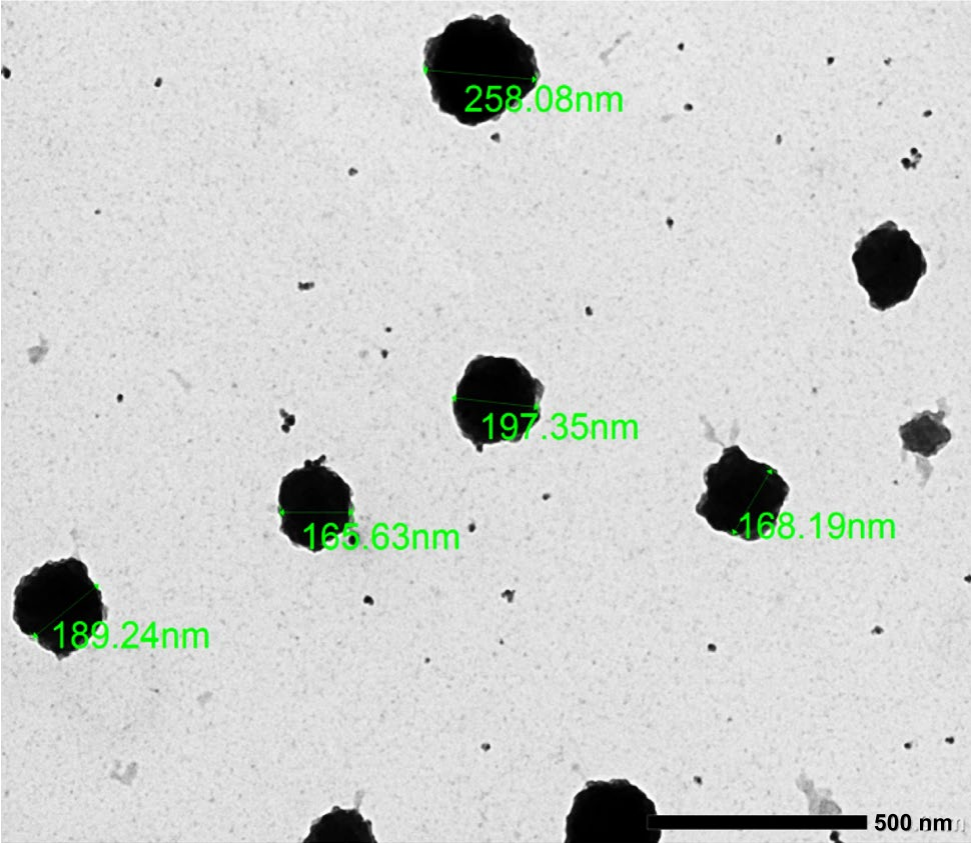
TEM micrograph of DAS/CXB-loaded BSA NPs showing their morphology

**Fig. 2:**
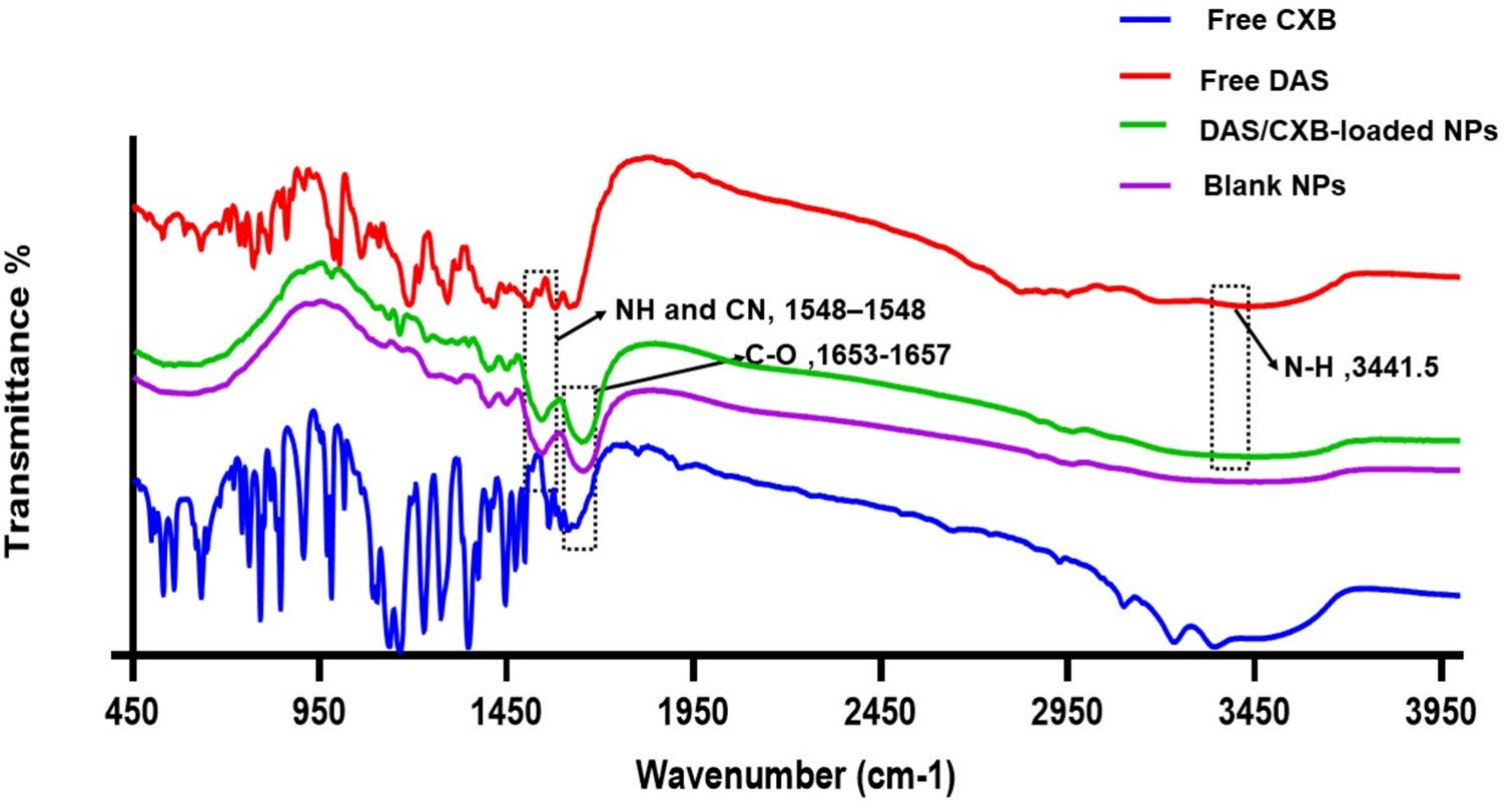
FT-IR spectra of free CXB, free DAS, DAS/CXB-loaded BSA NPs, and blank BSA NPs

To investigate the morphology and PS of the synthesized nano-formulation, transmission electron microscopy (TEM) was employed. As seen in Fig. 1, TEM micrographs showed that the DAS/CXB-loaded BSA NPs have a spherical shape and a PS of about 195.6 ± 33.45nm. As shown in Fig. 2, FT-IR spectra of both blank BSA NPs and DAS/CXB-loaded BSA NPs exhibited amide I peaks at 1657–1653 cm^−1^ owing to C-O stretching vibration and amide II peaks at 1548–1548 cm^−1^ attributable to NH bending in addition to CN stretching vibrations. The absence of any shift in peak positions in the spectrum indicated that there was no observable structural change in the protein following drug loading. But the peaks in DAS/CXB-loaded BSA NPs related to the presence of the 2 drugs were well observed. The spectrum of DAS displayed peaks at 3442 cm^−1^ attributable to NH stretching vibrations, at 3295 cm^−1^ owing to OH stretching vibrations, at 1619 cm^−1^ because of the CO stretching vibrations, at 1581 cm^−1^ attributable to C–C and CN stretching and at 1510 cm^−1^ because of the CHCH stretching vibrations. CXB spectrum displayed a peak at around 3337 cm^−1^ attributable to the NH2 group, peaks at 1614 cm^−1^ as well as 1562 cm^−1^ owing to NH stretching vibrations, also at 1349 cm^−1^ due to S = O stretching vibrations. Some of these peaks overlapped with the amide peaks of the NPs, but the characteristic peaks of DAS and CXB are still apparent, albeit at a lower intensity, in the spectra of DAS/CXB-loaded BSA NPs attributable to the low drug content. Furthermore, there was no significant shift in the distinctive peaks of both drugs in the dual drug-loaded NPs, indicating that they do not interact with the produced BSA NPs, which is necessary to maintain their efficacy.

**Fig. 3:**
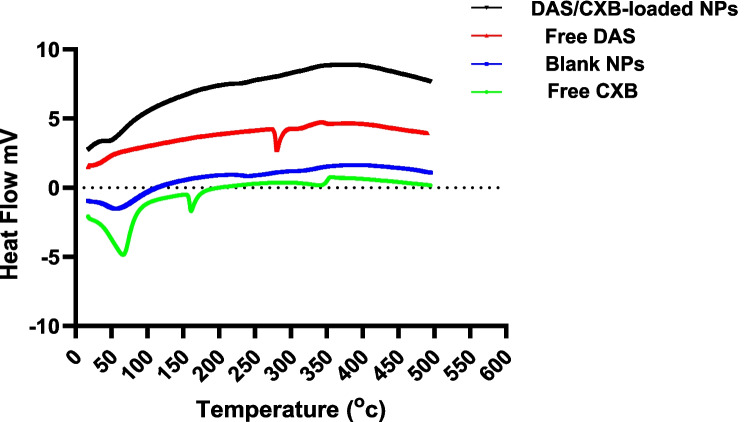
DSC thermograms of free DAS, free CXB, blank NPs and DAS/CXB-loaded NPs

Figure 3 showed the thermal behavior of BSA NPs loaded with DAS/CXB. The endothermic peaks for free DAS (283°C) as well as free CXB (160°C) were prominent. At 59 and 240°C, the blank BSA NPs' endothermic peaks were seen. DAS and CXB endothermic peaks disappeared in the dual-loaded BSA NPs, and the peak patterns of the DAS/CXB-loaded BSANPs resembled those of the BSA peaks. The absence of the peaks reflects the amorphous features of the albumin formula, while the peaks of BSA at 59 and 240°C shifted slightly, indicating the presence of an interaction between the drug and albumin, which might alter the molecular structure of the protein. In general, it is recognized that the compound's endothermic peaks could disappear or change due to the inclusion of the drug molecules into the vehicles (Sedov et al. 2020).

**Fig. 4:**
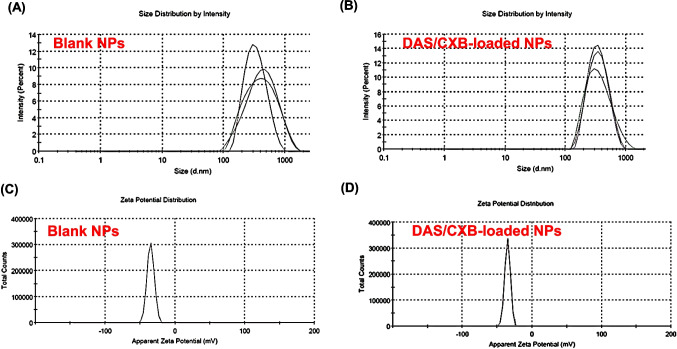
Physicochemical characteristics of the prepared NPs; hydrodynamic PS distribution of blank BSA NPs (**A**), hydrodynamic PS distribution of DAS/CXB-loaded BSA NPs (**B**), zeta potential distribution of Blank BSA NPs (**C**); zeta potential distribution of DAS/CXB-loaded BSA NPs (**D**)

As shown in Fig. 4A and B, the simultaneous encapsulation of DAS and CXB into the NPs caused a size decrease from 360.33 ± 7.58 in case of blank NPs to 336.6 ± 1.098 nm without a discernible change in the surface charge. This decrease in the particle size could be attributed to the possible interaction between drugs and albumin's hydrophobic binding sites (Sabra et al. 2019; Wang et al. 2023). The zeta potential is the main component in NPs' adsorption onto cell membranes and is crucial for their stability in suspension. The PS and zeta potential determine the endocytotic absorption rate upon adsorption. The results depicted that blank BSA NPs had a negative zeta potential of −34.8 ± 5.17 mV, while DAS/CXB-loaded BSA NPs had a zeta potential of −35 ± 4.03 mV, indicating that both preparations were stable without any aggregations and the encapsulation of drugs didn’t affect the charge of the NPs (Fig. 4C and D) (Tartari et al. 2023).

PDI can assess the average homogeneity of a particle solution. High PDI values indicate wide size distribution in the particle sample. Besides, PDI reveals NPs aggregation, as well as the uniformity and efficacy of particle surface changes across samples. A sample is deemed monodisperse if PDI is below 0.1, however, a PDI value above 0.4 and a value between 0.1 and 0.4 suggest that it is highly polydisperse and has a moderately scattered distribution, respectively. The value of PDI varies from 0.0 to 1.0. In practice, values of 0.2–0.3 or lower are generally considered acceptable for nanoparticle materials. The prepared formulations displayed PDI values of about 0.267 ± 0.021 and 0.211 ± 0.019 for blank and DAS/CXB-loaded BSA NPs, respectively (Supplementary Table S1).

**Fig. 5:**
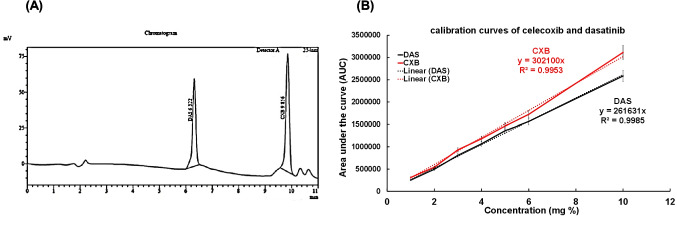
HPLC chromatograms of DAS/CXB combination standards at λ ◦ C for 15 min at 254 nm with mobile phase of acetonitrile and ammonium buffer (pH 11 (45:55 v/v)) (**A**) and the calibration curves of both DAS and CXB (**B**)

The encapsulation efficiency was evaluated using an HPLC system equipped with a diode array detector after validating the method for linearity, accuracy, and precision in the range of 0.00–10 mg% for both drugs via using a calibration curve for both drugs (Fig. 5A and B). The encapsulation efficiency (EE%) was found to be ≈ 97% for CXB and ≈ 95% for DAS, and the drug loading (DL%) was ≈ 8.84% for CXB and ≈ 8.68% for DAS.

### *In-vitro* drug release

**Fig. 6:**
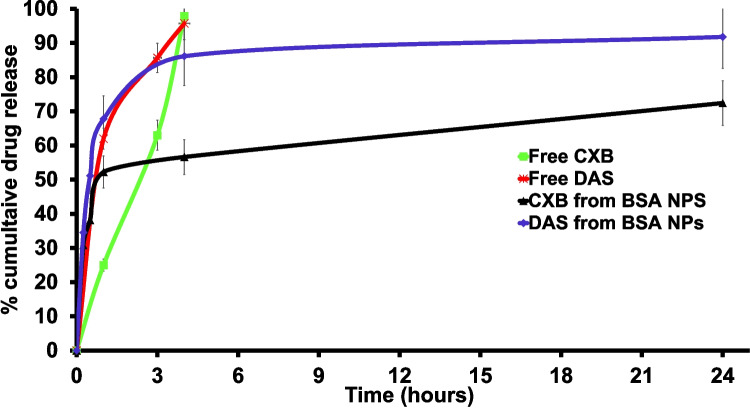
*In-vitro* drug release of DAS and CXB in acetate buffer supplemented with TritonX-100, pH 5.5 from DAS/CXB-loaded BSA NPs

To mimic the acidic conditions of the tumor microenvironment, the release patterns of both drugs were studied at pH 5.5 (Zhao et al. 2013) as earlier dissolution studies on DAS at pH 7.4 reported exceptionally poor solubility owing to its hydrophobicity, precluding direct contact with the dissolution medium as well as allowing DAS to float on the surface. In contrast, its solubility was enhanced in the acidic environment, particularly when surfactants as Triton X-100 are present, which has the ability to sustain the sink conditions during the experiment (Sabra et al. 2019). Samples were filtered, and the amount of DAS and CXB released was measured using HPLC. The sustained release of the drug from the NPs hinders its rapid metabolism and degradation (Langer and Wise 2019). As shown in Fig. 6, 85% of DAS and 55% of CXB were released after 4 h from the DAS/CXB-loaded BSA NPs. A fast initial release of both drugs from the NPs was evident. DAS was entirely released in 24 h, whereas 72% of CXB was released in the same time period. In the case of free drugs, they were almost completely released after 4 h.

### *In-vitro* cytotoxicity

**Fig. 7:**
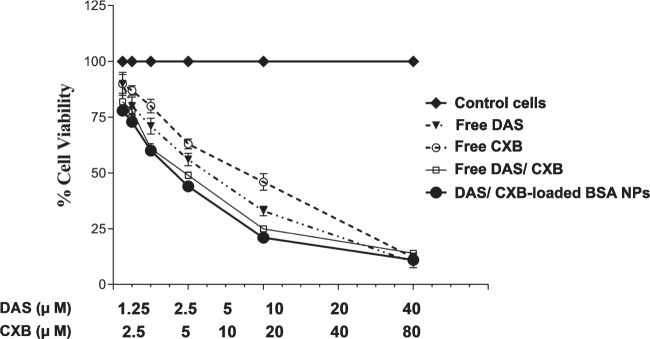
*In-vitro* cytotoxicity of different treatments against oral cancer cells after incubation with cells for 72 h at 37 °C. Data were shown as mean ± S.D. (*n* = 3)

Figure 7 and Table 1 display the IC50s, CIs, and DRIs. When combining free DAS with free CXB at the threshold of cytotoxicity (IC50), the cytotoxicity CI was 1.059 in SCC-4 cells, indicating an additive cytotoxic effect between DAS and CXB. However, when DAS was combined with CXB in the dual loaded nano-formulation at the threshold of cytotoxicity (IC50), the cytotoxicity CI was 0.858 in SCC-4 cells, indicating strong synergistic effect between the two drugs. The DRIs of DAS in the free combination and the dual loaded nano-formulation were 1.51 and 1.87 compared to free DAS. Similarly, DRIs of CXB in the free combination and the dual loaded nano-formulation were 2.49 and 3.07 compared to free CXB. Intriguingly, blank BSA NPs were found to be non-toxic to the cells at all the investigated concentrations, probably due to its remarkable biocompatibility as previously described (Li et al. 2012b).

**Table 1:** Combination indices (CIs) using the MTT assay of Dasatinib (DAS) and Celecoxib (CXB) combinations either free or in the dual loaded nano-formulation and Dose reduction indices (DRIs)

Drug/Combo	CI value	Total IC50 of the combination (μM)	DAS dose (μM)	CXB dose (μM)	Blank dose (μM)	DRI of Free DAS	DRI of Free CXB
Free DAS			11.2383 ± 1.82				
Free CXB				36.9129 ± 2.31			
Blank NPs					1940.58 ± 2.69		
FreeDAS/CXB Combination	1.05991	22.2106 ± 1.77	7.40352 ± 0.61 ^a^	14.8070 ± 1.161 ^c^		1.51796	2.49293
DAS/CXB Loaded NPs	0.85892	17.9988 ± 1.79	5.99961 ± 0.412 ^a,b^	11.9992 ± 1.368 ^c^		1.87317	3.07628

Values of CIs and DRIs of each drug were calculated using the CompuSyn software at IC_50_ following 48-h treatment of SCC-4 cells. IC50s were expressed as mean ± SD, (^a^) significant difference vs. free DAS alone, (^b^) significant difference vs. DAS in the free combination formula and (^c^) significant difference vs. free CXB alone

### *In-vitro* antitumor efficacy

#### Effect of the treatment regimen on COX-2 protein expression level in SCC-4 cell lysates

**Fig. 8:**
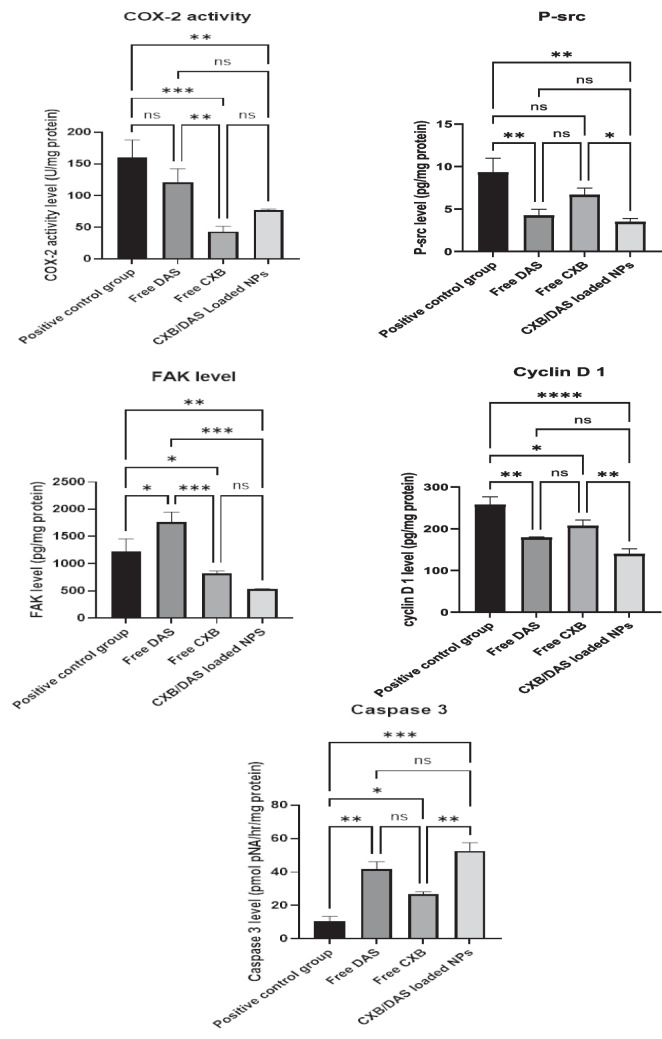
*In-vitro* antitumor efficacy showing the protein level of: **A** COX-2 activity; **B** p-Src; **C** FAK; **D** cyclin D1; and **E** Caspase 3

Figure 8A indicates that DAS/CXB-loaded BSA NPs inhibited COX-2 activity by 51.61% compared to the positive control (*p* = 0.0029) while free CXB had a greater effect on inhibiting COX-2 compared to the positive control, and free DAS-treated groups, respectively (72.8%, *p* = 0.0004 and 64.25%, *p* = 0.0073).

#### Effect of the treatment regimen on p-Src and FAK protein expression levels in SCC-4 cell lysates

Figure 8B infers that DAS/CXB-loaded BSA NPs decreased the p-Src protein expression level by 62.4%, and 47.6% in relation to the positive control, and free CXB-treated groups, respectively (*p* = 0.0012, and *p* = 0.0317). The results herein Fig. 8C demonstrate that DAS/CXB-loaded BSA NPs reduced the FAK protein expression level by 56.6%, 69.83% in relation to the positive control, free DAS-treated groups, respectively (*p* = 0.0021, and *p* = 0.0001).

#### Effect of the treatment regimen on cyclin-D1 protein expression level in SCC-4 cell lysates

Figure 8D shows that DAS/CXB-loaded BSA NPs decreased the level of cyclin D1 by 45.8%, and 32.67% in comparison to the positive control, and free CXB-treated groups, respectively (*p* =  < 0.0001, and *p* = 0.0028).

#### Effect of the treatment protocol on Caspase-3 protein expression level in SCC-4 cell lysates

Figure 8E depicts that DAS/CXB-loaded BSA NPs increased the protein expression level of Caspase-3 significantly by 397%, and 96.7% compared to the positive control, and free CXB-treated groups, respectively (*p* =  < 0.0001, and *p* = 0.0012).

## Discussion

Nanoparticles (NPs)-based drug delivery systems have demonstrated countless merits in cancer therapy, including improved pharmacokinetics, specific targeting of tumors, minimal adverse effects, and decreased therapy resistance (Yao et al. 2020). They ensure sustained drug release in addition to transporting the medications to their intended locations (Afzal et al. 2022). Nanocarriers can extend drug half-lives and induce drug accumulation in tumor tissues due to their size, surface properties, and enhanced permeability and retention, potentially improving cancer immunotherapy, ablation treatments, chemotherapy, and gene therapy (Giustarini et al. 2021).

Proteins and peptides are key areas of nanomedicine research, with protein nanoparticles being valuable for tissue engineering and drug delivery due to their: (a) Biocompatibility (Mahmoudi et al. 2023); (b) Biodegradability as these materials break down within the body, and the resulting amino acids are absorbed by the surrounding tissues to make proteins or energy; (c) The possibility of simple and inexpensive manufacture, given that proteins are found in all forms of nature and that a variety of proteins can be mass-produced using recombinant protein synthesis techniques; (d) High drug binding capacity, as proteins possess numerous functional groups that enable them to bind and transport significant amounts of drugs; and finally (e) Proper cell uptake (Ovais and Chen 2018).

Bovine serum albumin (BSA) is a water-soluble protein that noncovalently binds medications or inorganic compounds, allowing them to be delivered to various parts of the body. Its well-defined structure allows electrostatic adsorption of both positively and negatively charged molecules, allowing significant drug incorporation into these nanoparticles. Moreover, these nanoparticles have shown promise due to their non-immunogenicity, biocompatibility and site-specific delivery (Amighi et al. 2020).

The desolvation technique was chosen for preparing DAS/CXB-loaded BSA NPs due to its simplicity and control over PS and PDI by adjusting pH, ionic composition, and albumin solution concentration. Ethanol as a desolvating agent is preferred over others due to its easy control over Nanoparticles' size (Sadeghi et al. 2014). Moreover, the pH of the formulation was adjusted to be around 7.6 in order to be away from the isoelectric point of BSA which is nearly pI = 5.4, and hence preventing its agglomeration. Another reason for adjusting the pH above 6 is the the fact that BSA above its pI carries net negative charge, which in turn increases the repulsion force keeping the formed NPs well dispersed with smaller size (Tartari et al. 2023).

Nanoparticles' size and morphology influence release rate, drug solubility, stability, biocompatibility, and infiltration inside the tissues. Smaller nanoparticles stay in the circulation longer. The enhanced permeation and retention (EPR) effect takes place when the tumor vasculature leaks, allowing nanoparticles to penetrate and remain in the tumor bed. It was reported that the leaky tumor vasculature has PS between 380 and 780 nm. The EPR effect, reduced lymph outflow, and vascular hyperpermeability are important mechanisms for passively targeting NPs in solid tumors (Loureiro et al. 2016). DAS/CXB-loaded BSA NPs, with PS of 336.6 nm, can passively target oral cancer cells due to their sphere-shaped size and surface charge, relying on EPR effect for tumor targeting (Kunde and Wairkar 2022).

The in-vitro release profile of DAS was faster than CXB, possibly due to differences in binding and intramolecular interaction between the drugs and the NPs, and the varied compatibility of each drug with the NPs. These results could be attributed to the high binding affinity of all COX-2 inhibitors including CXB towards BSA with association constant of 10^4^–10^5^ with hydrophobic interactions and hydrogen bonding as the predominant interactions (Seedher and Bhatia 2009), which might delay the release of CXB. Regarding DAS, it displayed faster release in comparison to CXB due to the reported moderate binding affinity between DAS and BSA, which might provide a suitable release pattern of DAS from the BSA nanoparticles. Hydrogen bonding as well as van der Waal’s forces were the most important interaction forces and also the binding process was spontaneous, which could explain the faster release of DAS as compared to CXB (Naik and Jaldappagari 2019). Our study found that a weaker drug-NP interaction leads to a faster drug release profile. A recent study evaluating the release of CXB from BSA NPs revealed a comparable delay in the release of CXB from the BSA NPs (Kumar and Singh 2022). However, in another study encapsulating CXB in human serum albumin (HAS), the drug exhibited sustained release profile over 72 h with only 16% released, which was much lower than the observed release in our study. This might be related to the difference in the release conditions as the release medium in this study was 60 mL of PBS buffer, pH 7.4 (Khandelia et al. 2024). DAS was previously encapsulated in sodium caseinate micelles and zein-lactoferrin micelles with about 36% of the drug was released after 24 h in case of sodium caseinate micelles and 85% in case of zein-lactoferrin micelles in the same release conditions used in this study, indicating that the release profile of DAS is greatly dependent on the type of the protein used in the preparation of the nanocarrier. This might be related to the difference in the binding affinities of DAS towards different proteins (Sabra et al. 2019; Lamey et al. 2023).

A substantial body of research reported that COX/prostaglandin E2 (PGE2) pathway is implicated in the development as well as the progression of various cancers, including OSCC (Nasry et al. 2018). COX-2 is highly expressed in oral dysplastic lesions, OSCC tumors, and nearby lymphocytic infiltrates, and rarely expressed in normal epithelium (Erovic et al. 2003; Shibata et al. 2005; Pontes et al. 2013). Overexpression of COX-2 in OSCC triggers PGE2 release, which acts on prostaglandin E2 receptors on the cell surface to propel OSCC development (Abrahao et al. 2010). The COX-2/PGE2 axis in OSCC cells promotes migration and upregulates intercellular adhesion molecule-1 expression, and is related to OSCC metastases (Yang et al. 2010). It was reported that COX-2/PGE2 signaling can enhance cancer stemness (Rudnick et al. 2011; Li et al. 2012a; Thanan et al. 2012; Zhang et al. 2013), and impact the immune response to cancer cells by decreasing dendritic cell (DC) activity, reducing DC maturation, hindering antigen presentation, and activating T cells (Zhang et al. 2013). Additionally, it influences angiogenesis via VEGF, or can directly control endothelial cell proliferation (Gately and Li 2004). Interestingly, PGE2 can trigger hypoxia-inducible factor 1 alpha, leading to enhanced VEGF expression (Jung et al. 2003). Taken all together, COX-2/PGE2 signaling axis is linked to oxidative damage, inflammation, and epidermal growth factor receptor signaling, making targeting them a promising strategy for oral cancer treatment.

Supporting our findings, a previous study found that CXB might be effective in individuals at increased risk of developing OSCC, and in those who are in the early stages and do not have nodal metastases. In preclinical models, CXB significantly inhibited tumor proliferation in head and neck squamous cell carcinoma (Yip-Schneider et al. 2001), and in OSCC cells* in-vitro* (Shin et al. 2013). CXB inhibited FOXM1, a transcription factor that controls OSCC development and promotes angiogenesis, invasion, and metastasis (Lam et al. 2013). A 5-week CXB treatment significantly reduced OSCC tumor growth in a murine xenograft model, reversing EMT progression biomarkers like Snail suppression and cadherin switch initiation (Yang et al. 2006). 50 μM CXB treatment significantly increased E-cadherin gene expression and suppressed EMT transcriptional factors in OSCC cell lines (HSC-2 and HSC-4) after 12 h (Fujii et al. 2014).

Oral cancer progression is related to the overexpression of the Src/FAK axis, a key signaling cascade involved in cell proliferation, migration, invasion and metastasis (Raji et al. 2023). Src, positioned at the nexus of a multitude of important signal transduction cascades, is a versatile target. It interacts with FAK, enhancing cell proliferation, survival, and migration. FAK can also activate Src, sustaining the oncogenic signaling. Src and FAK promote EMT, contributing to poor oral cancer outcomes. Accordingly, targeting the Src/FAK axis shows potential as a therapeutic approach for treating oral cancer (Wu et al. 2022).

DAS inhibits Src family kinases (SFKs) via binding to the ATP-binding site, thus inhibiting Src phosphorylation at critical tyrosine residues and decreasing its activation and function in cancer progression signaling pathways. Upon DAS treatment, FAK phosphorylation at Y397 is changed, reducing FAK signaling, critical for cell adhesion and migration (Araujo and Logothetis 2010). Additionally, DAS reduces the phosphorylation of substrates associated with FAK, such as paxillin and p130 Crk-associated substrate (p130CAS), disrupting their anticipated roles in cell adhesion and motility (Caccia et al. 2010)**.** Taken all together, DAS can inhibit the capability of cells for invasion and migration by inhibiting the Src/FAK axis (Chan et al. 2012). Our findings herein are comparable with previous researches that reported that DAS suppressed Src activity (Shor et al. 2007; Buettner et al. 2008; Nautiyal et al. 2009).

Previous research on various breast cancer cell lines revealed that DAS substantially suppressed FAK phosphorylation (Pichot et al. 2009). Studies on hepatocellular carcinoma cell lines revealed that DAS might interact with various molecules to limit FAK phosphorylation, hence decreasing motility and invasion (Chang and Wang 2013). Research on hepatocellular carcinoma and nasopharyngeal cancer cell lines indicated that FAK is a down-stream effector of Src (Li et al. 2015). The study suggested a reciprocal catalytic activation mechanism between FAK and Src, as cell migration necessitates FAK activity, and FAK activation necessitates Src activity (Serna-Marquez et al. 2013). Similarly, CXB lowered FAK protein levels in this study, which aligns with previous findings (Casanova et al. 2008; Bai et al. 2009; Cai et al. 2017). Furthermore, the combination significantly reduced FAK protein level over the monotherapies in a previous study (Lamey et al. 2023).

Mounting data support the dysregulation of mammalian cyclins as well as cyclin dependent kinases (CDKs) in cancer as well as their oncogenic capacity in experimental animal models (Musgrove et al. 2011). Cyclin D1 is a key regulatory protein that governs cell cycle progression, particularly the G1-S phase transition. Cyclin D1 amplification and overexpression was evidenced in OSCC (Anjum et al. 2023). It was reported that cyclin D1 overexpression is associated with poor prognosis in OSCC, encompassing lymph node involvement, greater tumor size, progressive clinical stage, lower disease-free and overall survival, and lack of therapeutic responsiveness (Ramos-García et al. 2019). In this sense, Cyclin D1 is a promising target for oral cancer treatment, with ongoing research focusing on developing effective therapeutic strategies to combat OSCC. Notably, DAS showed substantial anti-proliferative effects on YD-38 and HSC-3 oral cancer cells via a range of tightly regulated mechanisms that collectively inhibit tumor development and progression. DAS arrests the cell cycle at the G1 phase by downregulating cyclins and CDKs thus hindering the transition of cancer cells through the cell cycle to subsequent phases. Additionally, DAS affects numerous signaling cascades via its ability to inhibit Src. In addition to its capacity to halt the phosphorylation of various receptors (e.g. EGFR, STAT-3, V-akt Murine Thymoma Viral Oncogene Homolog (AKT), and Extracellular signal-regulated kinase (ERK-1/2)) thus inhibiting crucial signaling cascades that ensures cell proliferation (Park et al. 2021). In addition, DAS has previously been shown to reduce proliferation in a plethora of studies conducted on various cancer types (Song et al. 2006; Nautiyal et al. 2009; Caccia et al. 2010; Le et al. 2010; Chang and Wang 2013; Li et al. 2015; Shindikar et al. 2016). DAS, with its anti-growth properties, could be an intriguing treatment option for oral cancer because it affects multiple targets and signaling cascades, warranting further exploration.

Pertaining to Celecoxib, it blocks signaling pathways that contribute to tumor proliferation via its ability to selectively inhibit COX-2 and reduce PGE2 production (Wen et al. 2020). Moreover, CXB halts the cell cycle at the G0/G1 phase by overexpressing various cyclin-dependent kinase inhibitors as p21 (Yoshida et al. 2019). It was reported that Celecoxib effectively delayed early oral cancer lesions and slowed tumor growth in preclinical models, with a dose-dependent reduction in tumor growth in hamster cheek pouch models (Feng and Wang 2006). Notably**,** CXB dramatically reduced tumor progression in a plethora of studies conducted on various cancer types (Yip-Schneider et al. 2001; Koki and Masferrer 2002; Denkert et al. 2003; Basu et al. 2005; Liu et al. 2012). Furthermore, the combination of CXB with DAS revealed a synergistic effect in HCT116 colorectal cancer cell line (Lamey et al. 2023).

Caspase-3 is a key effector caspase involved in both intrinsic and extrinsic apoptotic cascades. It triggers proteolytic processes, leading to morphological and biochemical alterations, including DNA fragmentation and membrane blebbing. Caspase-3 expression can be altered by genetic mutations, epigenetic alterations, or abnormal signaling cascades, allowing malignant cells to survive longer. Resistance to chemotherapy and radiotherapy reduces caspase-3 activation, highlighting its importance as a therapeutic target (Varışlı et al. 2023). DAS has substantial pro-apoptotic effect on oral cancer cells, specifically YD-38 and HSC-3 cell lines. It triggers apoptosis via the intrinsic pathway by activating critical apoptotic regulators, as caspase-3 and caspase-9. Its action involves downregulating Mcl-1, an anti-apoptotic protein that enables the cancer cells to evade apoptosis. DAS encourages endoplasmic reticulum stress, by increasing glucose-regulated Protein 78 expression and the phosphorylation of eukaryotic translation initiation factor 2A, suggesting that ER stress is a key mediator in DAS-induced apoptosis. Furthermore, DAS has been shown to enhance caspase-3 activation in numerous studies (Le et al. 2010; Lin et al. 2012; Mpakou et al. 2013; Song et al. 2013; Che et al. 2015; Li et al. 2015). Celecoxib promotes apoptosis via both COX-dependent and COX-independent pathways. CXB disturbs the mitochondrial membrane potential, thus releasing cytochrome c and activating caspases, particularly caspase-9 and caspase-3 (Ding et al. 2005). In addition to this, CXB downregulates anti-apoptotic proteins such as Mcl-1 and Bcl-2, further amplifying its apoptotic effects (Liu et al. 2012).CXB’s ability to modulate inflammatory responses can lead to enhanced apoptosis in inflamed tissues, such as those affected by chronic periodontitis associated with OSCC (Yoshida et al. 2019). Notably, CXB treatment has been shown to significantly increase caspase-3 levels herein, consistent with findings from several other studies (Koki and Masferrer 2002; Kim et al. 2004; Wang et al. 2017; Alian et al. 2024). The combination therapy significantly enhanced caspase-3 activation, surpassing the effects of each drug alone in previous studies (Moussa et al. 2021; Lamey et al. 2023).

## Conclusion

Protein polymers have shown increased potential as drug delivery systems for cancer therapy owing to their biocompatibility and low immunogenicity. Bovine serum albumin (BSA) is one among these natural proteins which is abundantly found in the blood circulation and is normally involved in the delivery of various active components, and hence it is an attractive candidate for the synthesis of nanoparticles to encapsulate and deliver hydrophobic drugs to oral cancer based on the enhanced permeability and retention (EPR) phenomenon. In this study, dasatinib (DAS; a tyrosine kinase inhibitor) and celecoxib (CXB; COX-2 inhibitor) were co-encapsulated into BSA nanoparticles using the desolvation technique. Dual drug-loaded NPs exhibited particle size of about 336.6 nm and zeta potential of about −35 mV. The in vitro drug release study showed sustained release of both drugs from the NPs in comparison to their free forms with faster release of DAS compared to CXB which might be attributed to the moderate binding affinity of DAS to albumin. In addition, the in vitro cytotoxicity study on SSC-4 human oral cancer cells revealed enhanced cytotoxic effect with lower IC50 and synergistic effect. Moreover, these nanoparticles showed improved in vitro antitumor efficacy in terms of reduced COX-2, cyclin D1, p-Src and FAK expression levels, associated with increased caspase-3 level, suggesting that these NPs can afford better drug internalization and sensitization to SSC-4 cells leading to improved outcomes. In the future, the preclinical potential of these NPs can be examined in vivo. Yet, an important issue still needs to be addressed including scaling up of these NPs with reproducible features and passing with all the formulation approval stages.

## Supplementary Information

Below is the link to the electronic supplementary material.

Supplementary file1 (DOCX 917 KB)

## Data Availability

All source data for this work (or generated in this study) are available upon reasonable request.
