# Supplementary material for: Bovine serum albumin nanoparticles encapsulating Dasatinib and Celecoxib for oral cancer: Preparation, characterization, and in-vitro evaluation
Source: Naunyn Schmiedebergs Arch Pharmacol. 2025 Feb 12;398(7):9291–306. doi: 10.1007/s00210-025-03829-1 (PMC12263490; doi:10.1007/s00210-025-03829-1)
Supplement: Supplementary file 1 — Supplementary file1 (DOCX 917 KB) [file 210_2025_3829_MOESM1_ESM.docx]

**Supplementary materials**

Figure S1: Fourier transform infrared spectra of free DAS, free CXB, blank NPs and DAS/CXB-loaded NPs.

Figure S2: DSC thermograms of free DAS, free CXB, blank NPs and DAS/CXB-loaded NPs.

Supplementary Table S1: Summary of three records for blank NPs and dual drug-loaded NPs.
